# Choroidale Neovaskularisation infolge einer „punctate inner choroidopathy“, dargestellt mittels optischer Kohärenztomographie-Angiographie

**DOI:** 10.1007/s00347-020-01200-8

**Published:** 2020-08-07

**Authors:** Martin Dominik Leclaire, Christoph R. Clemens, Nicole Eter, Nataša Mihailovic

**Affiliations:** Klinik für Augenheilkunde, Universitätsklinikum Münster, Domagkstr. 15, 48149 Münster, Deutschland

**Keywords:** White-Dot-Syndrome, Uveitis, Chorioretinitis, OCT‑A, CNV, White dot syndrome, Uveitis, Chorioretinitis, OCT‑A, CNV

## Abstract

Die „punctate inner choroidopathy“ (PIC) geht nicht selten mit der Ausbildung einer choroidalen Neovaskularisation (CNV) einher. Die Identifizierung einer frischen CNV im Rahmen einer PIC ist häufig schwierig. Wir präsentieren einen Fall einer 30-jährigen Patientin mit morphologisch typischer PIC. Eine CNV konnte weder in der optischen Kohärenztomographie (OCT) noch in der Fluoreszeinangiographie (FAG) sicher identifiziert werden. Die OCT-Angiographie (OCT-A) hingegen zeigte eine umschriebene CNV. Der Fall lässt eine hohe Dunkelziffer von nicht diagnostizierten, klinisch stummen und nicht therapiebedürftigen sekundären CNVs im Rahmen von PIC vermuten.

## Abstract (en)

Punctate inner choroidopathy (PIC) is often accompanied by the development of choroidal neovascularization (CNV). The identification of a fresh CNV in the context of PIC is often difficult. We present the case of a 30-year-old female patient with typical morphological features of PIC. A CNV could not be detected with certainty by optical coherence tomography (OCT) or by fluorescein angiography (FAG); however, OCT angiography (OCT-A) revealed a circumscribed CNV. The case suggests that there are a high number of undiagnosed, subclinical secondary CNVs not requiring treatment in PIC patients.

## Anamnese und klinischer Befund

**Figure 1:**
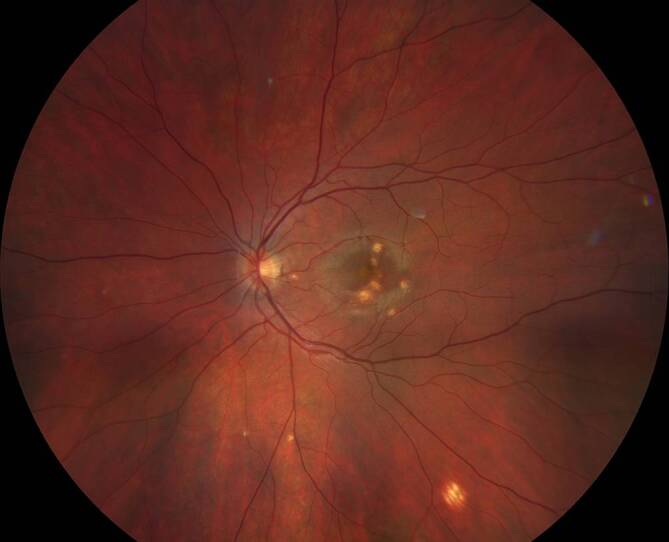

**Figure 2:**
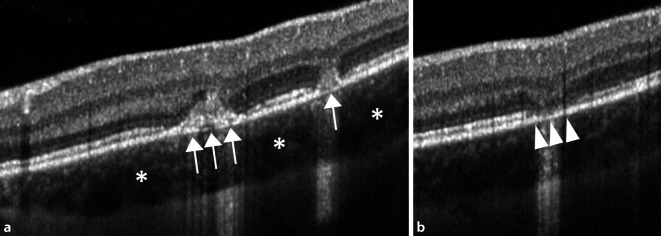

**Figure 3:**
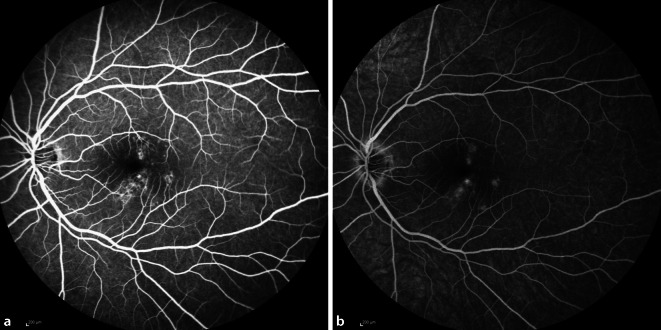

**Figure 4:**
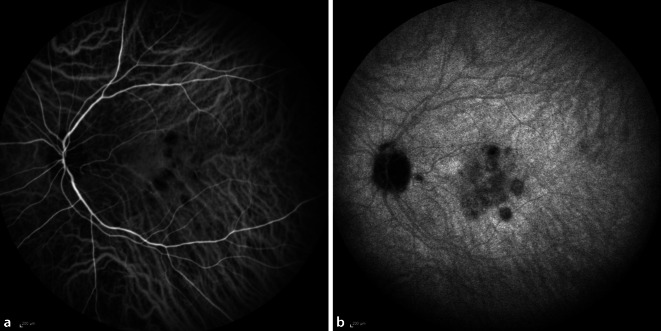

**Figure 5:**
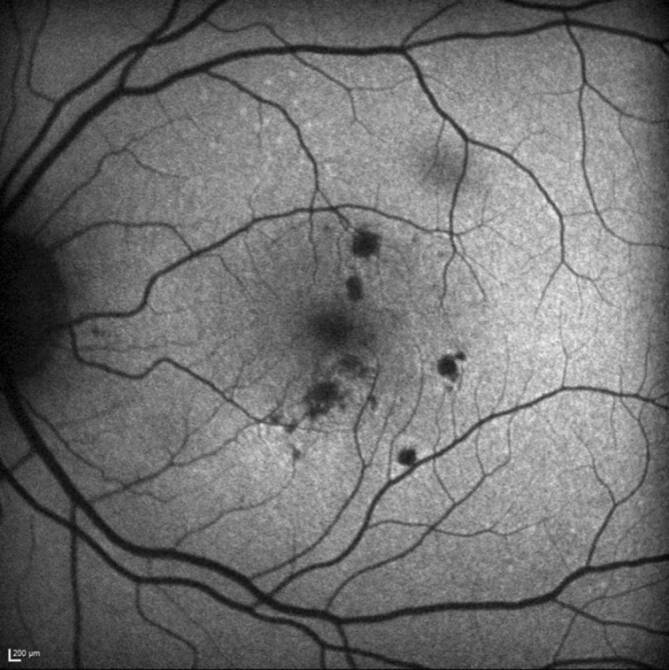

**Figure 6:**
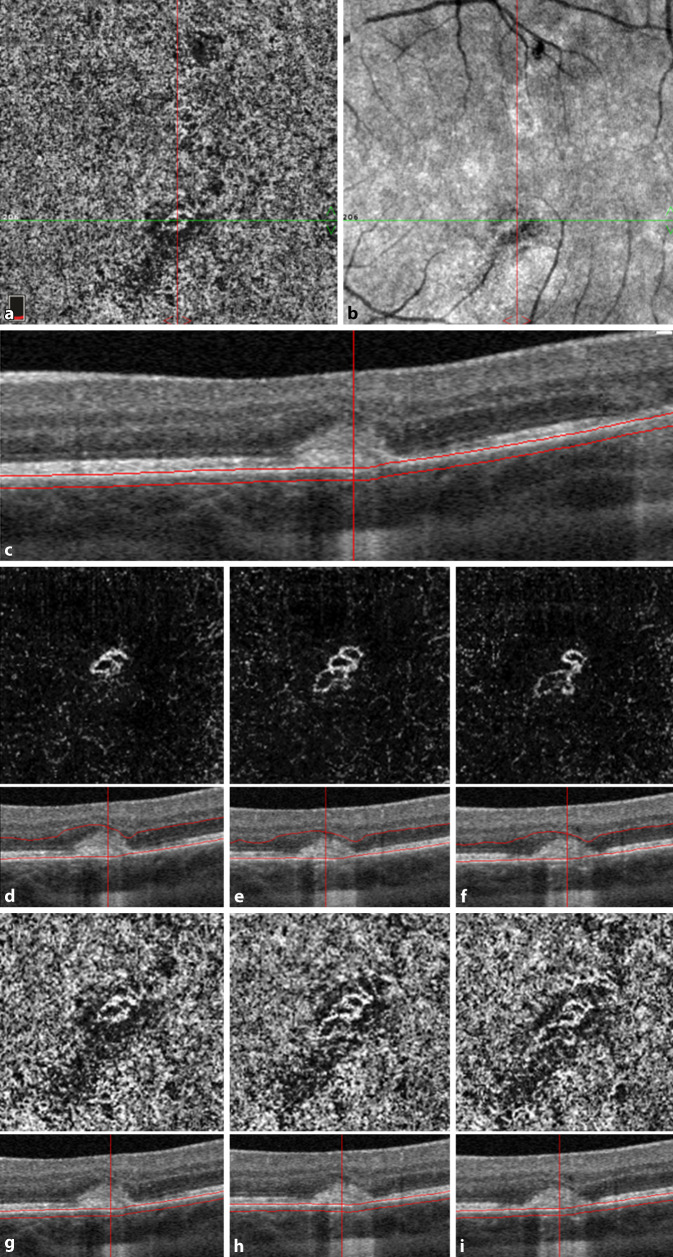

Eine 30-jährige, gesunde Patientin stellte sich mit seit 2 Wochen zunehmender Visusminderung am linken Auge und Wahrnehmung parazentraler grauer Flecken vor. Anamnestisch sei eine vergleichbare Symptomatik bereits 9 Monate zuvor aufgefallen. Zusätzlich berichtet die Patientin von 2 intravitrealen Injektionen, die sie in der Vergangenheit an dem betroffenen Auge erhalten hatte. Der bestkorrigierte Visus betrug beidseits 1,0 (−8,00 Sphäre rechts/−7,75 Sphäre links). Spaltlampenbiomikroskopisch zeigten sich reizfreie und klare optische Medien. Fundoskopisch imponierten am linken Auge multiple zentrale und vereinzelte mittelperiphere blass-gelbe chorioretinale Herde (Abb. 1). Diese korrelierten in der „spectral domain“ optischen Kohärenztomographie(SD-OCT)-Bildgebung mit einem hyperreflektiven Signal im Bereich der äußeren Körnerschicht und äußeren Grenzmembran und einer Atrophie der Photorezeptorbanden. Zusätzlich erschien die Choriokapillaris verdickt (Abb. 2a, b). Während in der Fluoreszeinangiographie (FAG) im Bereich der Läsionen eine Hyperfluoreszenz mit dezenter Leckage in der Spätphase festgestellt wurde (Abb. 3a, b), wiesen die chorioretinalen Herde in der Indocyaningrünangiographie eine Hypofluoreszenz auf (Abb. 4a, b). Im Bereich der Läsionen erschien das Autofluoreszenzsignal ausgelöscht (Abb. 5). Eine sekundäre choroidale Neovaskularisation (CNV) wurde mittels der beschriebenen Verfahren nicht detektiert. Erst in der OCT-Angiographie (OCT‑A, RT Vue XR Avanti, *Optovue Inc.*, Fremont, Kalifornien, USA) stellte sich im Bereich der inferioren Makula eine umschriebene CNV dar (Abb. 6a–i).

## Diagnose

In der Zusammenschau ergab sich die Diagnose einer „punctate inner choroidopathy“ (PIC) links mit einer inaktiven sekundären CNV.

## Therapie und Verlauf

Wir initiierten eine orale körpergewichtsadaptierte Kortikosteroidtherapie (Prednisolon 1 mg/kg Körpergewicht mit wochenweiser Reduktion über insgesamt 7 Wochen). Aufgrund fehlender Hinweise einer CNV-Aktivität wurde auf eine intravitreale Anti-vascular-endothelial-growth-factor(VEGF)-Therapie zunächst verzichtet. Eine Woche später zeigten sich die Läsionen umschriebener und regredient. Es bestand weiterhin kein Makulaödem bei stabilem Visus. Die anschließenden Kontrollen ergaben eine zunehmende Abgrenzbarkeit der chorioretinalen Herde mit fortschreitender Vernarbungstendenz bei insgesamt unveränderter Blutflussdetektion im Bereich der sekundären CNV (Abb. 6d–i).

## Diskussion

Die PIC ist eine Form der posterioren Uveitis und zählt zu den White-dot-Syndromen (WDS). Die Erkrankung wurde erstmals 1984 von Watzke et al. [10] beschrieben und betrifft typischerweise junge myope Frauen [3]. Der Anteil weiblicher Betroffener wird in einer größeren Studie mit 136 eingeschlossenen Patienten mit 93 % angegeben bei einem Durchschnittsalter von 36 Jahren und einer mittleren Myopie von −4,5 dpt [4]. Die Erkrankung kann sowohl uni- als auch bilateral auftreten, wobei eine bilaterale Affektion etwas häufiger auftritt. [4]. Glaskörper- oder Vorderkammerbeteiligung sind im Rahmen einer PIC typischerweise nicht nachweisbar [3]. Fundoskopisch imponieren multiple gelblich-weiße chorioretinale Läsionen von ca. 100–300 µm Größe. In 40–76 % der Fälle ist das Krankheitsbild mit der Entwicklung einer sekundären CNV assoziiert [9]. Die Wirksamkeit von intravitrealen Injektionen mit Anti-VEGF bei sekundärer CNV im Rahmen einer PIC ist in der Literatur beschrieben [2, 11]. Mit Ranibizumab steht inzwischen ein zugelassener Wirkstoff für seltene CNV-Ätiologien zur Verfügung [6].

Mittels systemischer Kortikosteroidtherapie wird in Einzelfällen ein Rückgang der Entzündungsherde festgestellt [8]. Es wird postuliert, dass sich eine systemische Kortikosteroidtherapie auch günstig auf die Visusentwicklung im Falle einer CNV auswirken kann [5].

Die Identifizierung einer frischen CNV bei aktiven Entzündungsprozessen im Rahmen der Erkrankung stellt in zahlreichen Fällen eine Herausforderung dar, da sich die chorioretinalen Entzündungsherde in der FAG ebenso wie eine sekundäre CNV mit konsekutiver Aufhebung der Blut-Retina-Schranke hyperfluoreszent darstellen. Ferner kann es sowohl bei einer CNV als auch bei einer aktiven PIC-Läsion zu Leckagen kommen [7]. Auch mittels OCT ist die Abgrenzung zwischen einer CNV und einer aktiven entzündlichen Läsion nicht eindeutig möglich, da sich sowohl eine CNV als auch eine aktive Läsion durch eine Auftreibung im Bereich der äußeren Netzhautbanden präsentieren können [1].

Dass die OCT‑A zur Detektion einer inaktiven sekundären CNV im Rahmen einer PIC der OCT-Untersuchung überlegen ist, konnten Levison et al. zeigen. Sie untersuchten 12 Patienten mit PIC oder der eng verwandten multifokalen Chorioiditis mittels OCT‑A und konnten in 15 von 17 Augen eine CNV darstellen, während nur in 3 Fällen kohärenztomographisch ein gesichertes Makulaödem nachgewiesen werden konnte [7].

Der hier präsentierte Fall stützt diese Ergebnisse. Auch bei unserer Patientin waren die Gefäßveränderungen in der konventionellen FAG und in der SD-OCT nicht sichtbar und lediglich mittels OCT‑A darstellbar. Die OCT‑A ist eine einfach durchzuführende und nichtinvasive Bildgebungsmodalität, mit deren Hilfe eine sekundäre CNV im Rahmen einer PIC frühzeitig erkannt werden kann. In einem Fall wie dem hier geschilderten sollten engmaschige Verlaufskontrollen stattfinden, um die Entstehung eines Makulaödems rechtzeitig erkennen und ggf. behandeln zu können. Der Patient sollte über die Möglichkeit der Entstehung eines Makulaödems aufgeklärt werden und bei Symptomen (wie etwa Metamorphopsien) eine zeitnahe Wiedervorstellung angeraten werden.

In Zusammenschau lässt sich eine hohe Dunkelziffer von bis dato nicht diagnostizierten sekundären CNVs im Rahmen einer PIC vermuten.

## Zusammenfassung

Die PIC ist mit einem erhöhten Risiko für die Entwicklung einer sekundären CNV verbunden, was eine Therapie mittels intravitrealer Anti-VEGF-Injektionen notwendig machen kann. Der hier vorgestellte Fall verdeutlicht, dass die OCT‑A eine vielversprechende Bildgebungsmodalität zur Identifikation einer sekundären CNV bei PIC darstellt.
